# Supplementary material for: Var∣Decrypt: a novel and user-friendly tool to explore and prioritize variants in whole-exome sequencing data
Source: Epigenetics Chromatin. 2023 Jun 14;16:23. doi: 10.1186/s13072-023-00497-4 (PMC10265870; doi:10.1186/s13072-023-00497-4)
Supplement: Supplementary file 1 — Additional file 1: Exome-seq variant analysis pipeline. [file 13072_2023_497_MOESM1_ESM.doc]

# **VarDecrypt: a novel and user-friendly tool to explore and prioritize variants in whole-exome sequencing data**

Mohammad Salma^1,2,*^, Elina Alaterre^4^, Jérôme Moreaux^3,4,5^, and Eric Soler^1,2,*^

^1^ Institut de Génétique Moléculaire de Montpellier, Univ Montpellier, CNRS, Montpellier, France

^2^ Université de Paris, Laboratory of Excellence GR-Ex

^3^ Department of Biological Hematology, CHU Montpellier, Montpellier, France;

^4^ Institute of Human Genetics, UMR 9002 CNRS-UM, Montpellier, France;

^5^ Institut Universitaire de France (IUF);

* correspondance :

MS : [mohammad.salma@igmm.cnrs.fr](mailto:mohammad.salma@igmm.cnrs.fr)

ES : [eric.soler@igmm.cnrs.fr](mailto:eric.soler@igmm.cnrs.fr)

## Additional file 1

## Exome-Seq analysis pipeline

To provide an all-in-one solution, we first implemented an Exome-seq variant analysis pipeline. The different steps are managed by Snakemake platform [1] which is a tool to create reproducible and scalable data analysis workflow (**Supp Fig.1**). It can be scaled to server, cluster, grid and cloud environment with no need to change the workflow definition. The WES analysis pipeline contains all needed tools within a Singularity and docker container (see **Supp Fig.1**). It consists in the following steps: first, the reads are aligned to the human genome using BWA-MEM [2] which is one of the most widely used well performing aligner for WES data. Post-mapping optimization is performed using GATK [3–6] suite which includes many tools to manage WES and Whole genome sequencing (WGS) data. For the variant calling step, the HaplotypeCaller is used for germline variants and Mutect2 for somatic ones from GATK4 [6] as recent studies have shown the efficiency of these tools in different scenarios [7–9]. After the variant calling procedure, the different variants need to be subsequently annotated. To this aim, we used the popular and powerful ANNOVAR tool [10] which integrates several useful databases (for example, dbSNP, 1000 genomes and Clinvar annotations databases). Our pipeline comes with a python master script (run_preVarDecrypt.py) which allows users to run the pipelines’ steps using one command line. It requires two config files: 1) a first one containing the path to fastq files and some optional parameters, 2) the second is a tabulated file to make the association between tumoral and control samples. To allow users preparing VarDecrypt inputs starting from VCF files, we provide a shorter pipeline to annotate and group samples’ data (**Supp Fig.1**).

Additional references:

[1] Köster J, Rahmann S. Snakemake—a scalable bioinformatics workflow engine. *Bioinformatics* 2012; 28: 2520–2522.

[2] Li H. Aligning sequence reads, clone sequences and assembly contigs with BWA-MEM. *ArXiv13033997 Q-Bio*, http://arxiv.org/abs/1303.3997 (2013, accessed 4 December 2020).

[3] McKenna A, Hanna M, Banks E, et al. The Genome Analysis Toolkit: A MapReduce framework for analyzing next-generation DNA sequencing data. *Genome Res* 2010; 20: 1297–1303.

[4] DePristo MA, Banks E, Poplin R, et al. A framework for variation discovery and genotyping using next-generation DNA sequencing data. *Nat Genet* 2011; 43: 491–498.

[5] Auwera GAV der, Carneiro MO, Hartl C, et al. From FastQ Data to High-Confidence Variant Calls: The Genome Analysis Toolkit Best Practices Pipeline. *Curr Protoc Bioinforma* 2013; 43: 11.10.1-11.10.33.

[6] Poplin R, Ruano-Rubio V, DePristo MA, et al. Scaling accurate genetic variant discovery to tens of thousands of samples. *bioRxiv* 2018; 201178.

[7] Xiao W, Ren L, Chen Z, et al. Toward best practice in cancer mutation detection with whole-genome and whole-exome sequencing. *Nat Biotechnol* 2021; 39: 1141–1150.

[8] Cornish A, Guda C. A Comparison of Variant Calling Pipelines Using Genome in a Bottle as a Reference. *BioMed Research International* 2015; 2015: e456479.

[9] Hwang S, Kim E, Lee I, et al. Systematic comparison of variant calling pipelines using gold standard personal exome variants. *Sci Rep* 2015; 5: 17875.

[10] Wang K, Li M, Hakonarson H. ANNOVAR: functional annotation of genetic variants from high-throughput sequencing data. *Nucleic Acids Res* 2010; 38: e164.
