# Supplementary material for: Var∣Decrypt: a novel and user-friendly tool to explore and prioritize variants in whole-exome sequencing data
Source: Epigenetics Chromatin. 2023 Jun 14;16:23. doi: 10.1186/s13072-023-00497-4 (PMC10265870; doi:10.1186/s13072-023-00497-4)
Supplement: Supplementary file 5 — Additional file 5: Table S4. Performance of pre Var∣Decrypt annotate vcf pipeline using two different methods of deployment. All times are in seconds. [file 13072_2023_497_MOESM5_ESM.docx]

| **Supp Table 4.** Performance of pre Var⏐Decrypt annotate vcf pipeline using two different methods of deployment. All times are in seconds. | | | |
| --- | --- | --- | --- |
| environment specification | Processes | Somatic Variant | Somatic Variant |
|  |  | input data size: | input data size: |
|  |  | vcf.gz = 7.96 GB | vcf.gz =36.8 MB |
|  |  |  |  |
|  |  | Samples: 59 | Samples: 22 |
| **OS:** Debian 5.10.46-1 x86_64  **Cluster:** slurm **CPU number:** 16 (2.20GHz) **Runtype:** Code Source | annotation step | 2601 | 520 |
|  |  |  |  |
|  | Group and Summary samples data step | 163 | 3 |
|  |  |  |  |
| **OS:** Debian 5.10.46-1 x86_64  **Cluster:** slurm **CPU number:** 16 (2.20GHz) **Runtype:** Singularity | annotation step | 1653 | 350 |
|  |  |  |  |
|  | Group and Summary samples data step | 151 | 9 |
|  |  |  |  |
