# Supplementary material for: Var∣Decrypt: a novel and user-friendly tool to explore and prioritize variants in whole-exome sequencing data
Source: Epigenetics Chromatin. 2023 Jun 14;16:23. doi: 10.1186/s13072-023-00497-4 (PMC10265870; doi:10.1186/s13072-023-00497-4)
Supplement: Supplementary file 6 — Additional file 6: Table S5. Performance of Var∣Decrypt after loading two types of input date in different operating systems with two different methods of deployment. All times are in seconds. [file 13072_2023_497_MOESM6_ESM.docx]

| **Supp Table 5.** Performance of Var⏐Decrypt after loading two types of input date in different operating systems with two different methods of deployment. All times are in seconds. | | | |
| --- | --- | --- | --- |
|  |  |  |  |
| environment specification | Processes | Somatic Variant  (new analysis) | Somatic Variant  (load processed data) |
|  |  | input data size: | input data size: |
|  |  | Tumoral_AllSamples.csv = 1.3 MB | Rdata file = 68.6 MB |
|  |  | Summary_allMaf.txt = 33.8 MB |  |
|  |  | Samples: 59 | Samples: 59 |
| OS: macOS Monterey MacBook pro 2020  Processor: Apple Silicon M1 RAM: 16 GB Runtype: code source | load input files | 1 | 1 |
|  | open and process data | 23.38 | 12.24 |
|  | filter by database | 1 | 1 |
|  | filter by the number of variant by gene | 2 | 2 |
|  | perform enrichement analyses | 89.59 | na |
| OS: macOS Monterey MacBook pro 2020  Processor: Apple Silicon M1 RAM: 16 GB Runtype: Docker | load input files | 1 | 1 |
|  | open and process data | 20.45 | 6.36 |
|  | filter by database | 1 | 1 |
|  | filter by the number of variant by gene | 2 | 2 |
|  | perform enrichement analyses | 67.34 | na |
| OS: Ubuntu (virtual machines in cloud) Processor: Processor: 2GHz AMD RAM: 8 GB Runtype: Docker | load input files | 2 | 4 |
|  | open and process data | 49.40 | 14.24 |
|  | filter by database | 2 | 2 |
|  | filter by the number of variant by gene | 3 | 4 |
|  | perform enrichement analyses | 134.61 | na |
