# Supplementary figures and images for: Var∣Decrypt: a novel and user-friendly tool to explore and prioritize variants in whole-exome sequencing data
Source: Epigenetics Chromatin. 2023 Jun 14;16:23. doi: 10.1186/s13072-023-00497-4 (PMC10265870; doi:10.1186/s13072-023-00497-4)

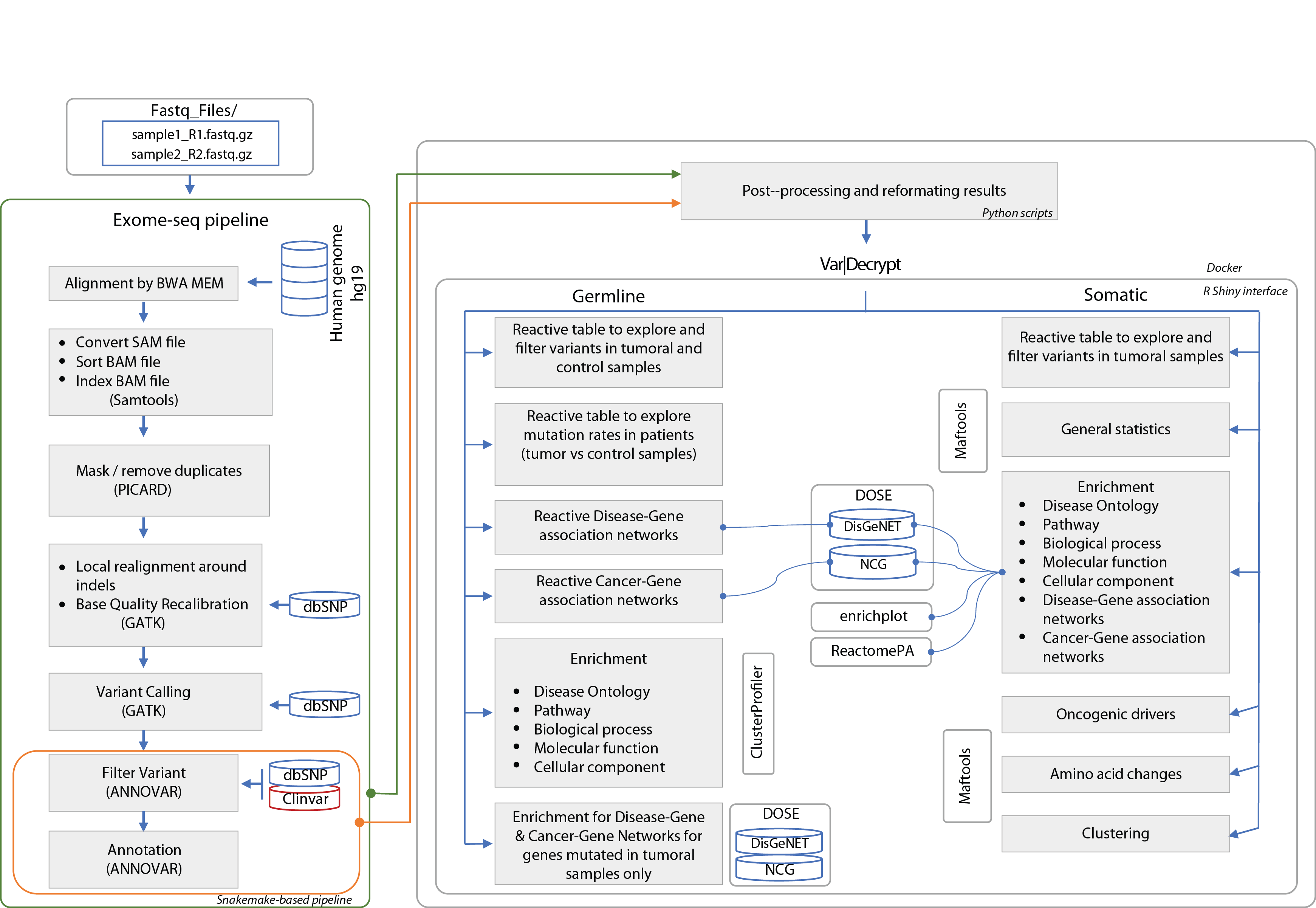

Supplement: Supplementary file 7 — Additional file 7: Figure S1. Bioinformatic pipelines used to process whole exome sequencing data. The WES and Var|Decrypt pipelines are depicted on the left and right, respectively. The various filtering steps and packages used are indicated. Two versions of the WES pipeline are available (The part highlighted in green corresponds to the pipeline to process WES data starting from fastq files; in orange the one allowing users todirectly processing vcf files). [file 13072_2023_497_MOESM7_ESM.png]

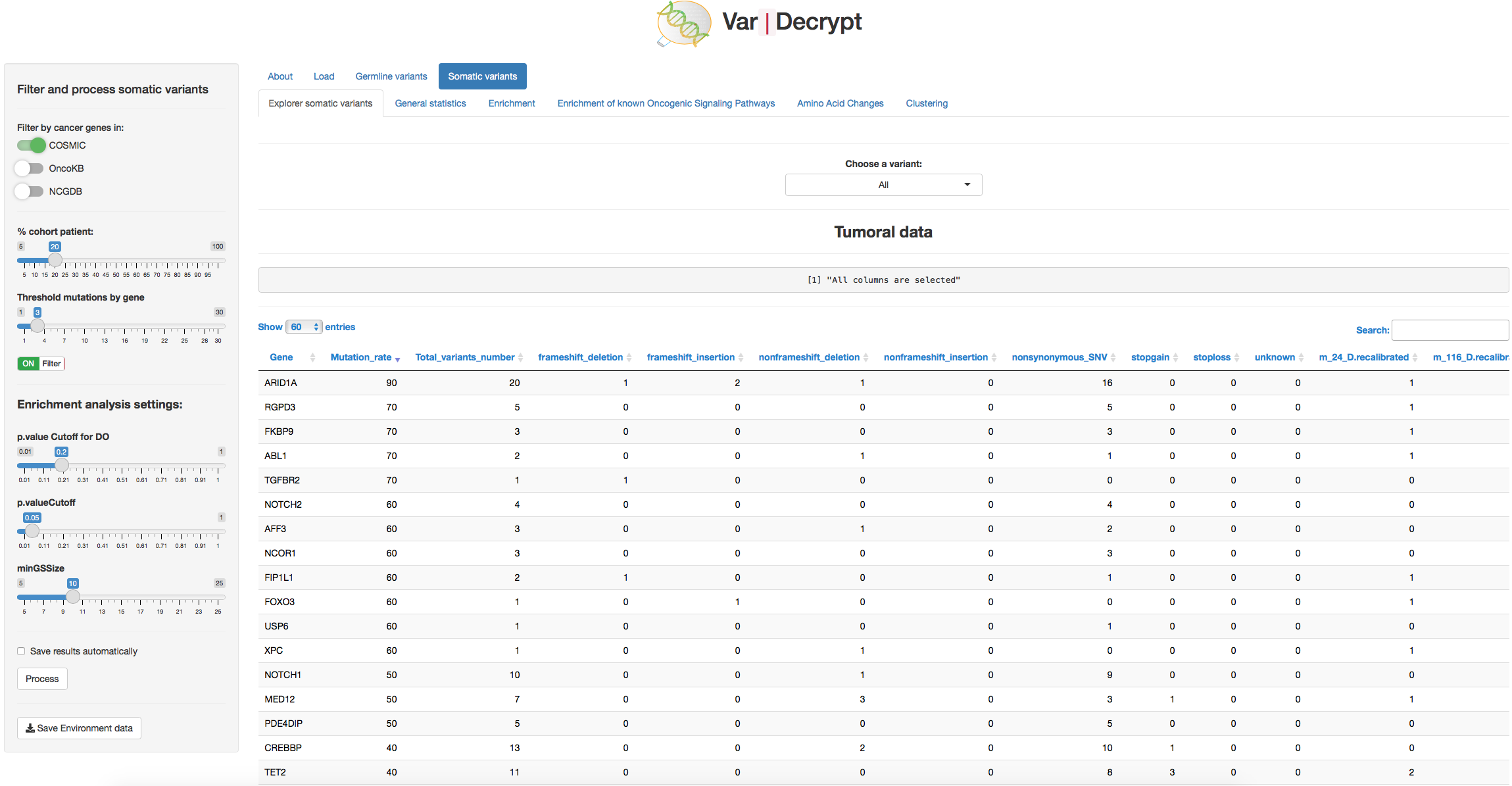

Supplement: Supplementary file 8 — Additional file 8: Figure S2. Example of Var|Decrypt front page showing the mutated gene list together withthe frequencies in the cohort and the mutation types. [file 13072_2023_497_MOESM8_ESM.png]

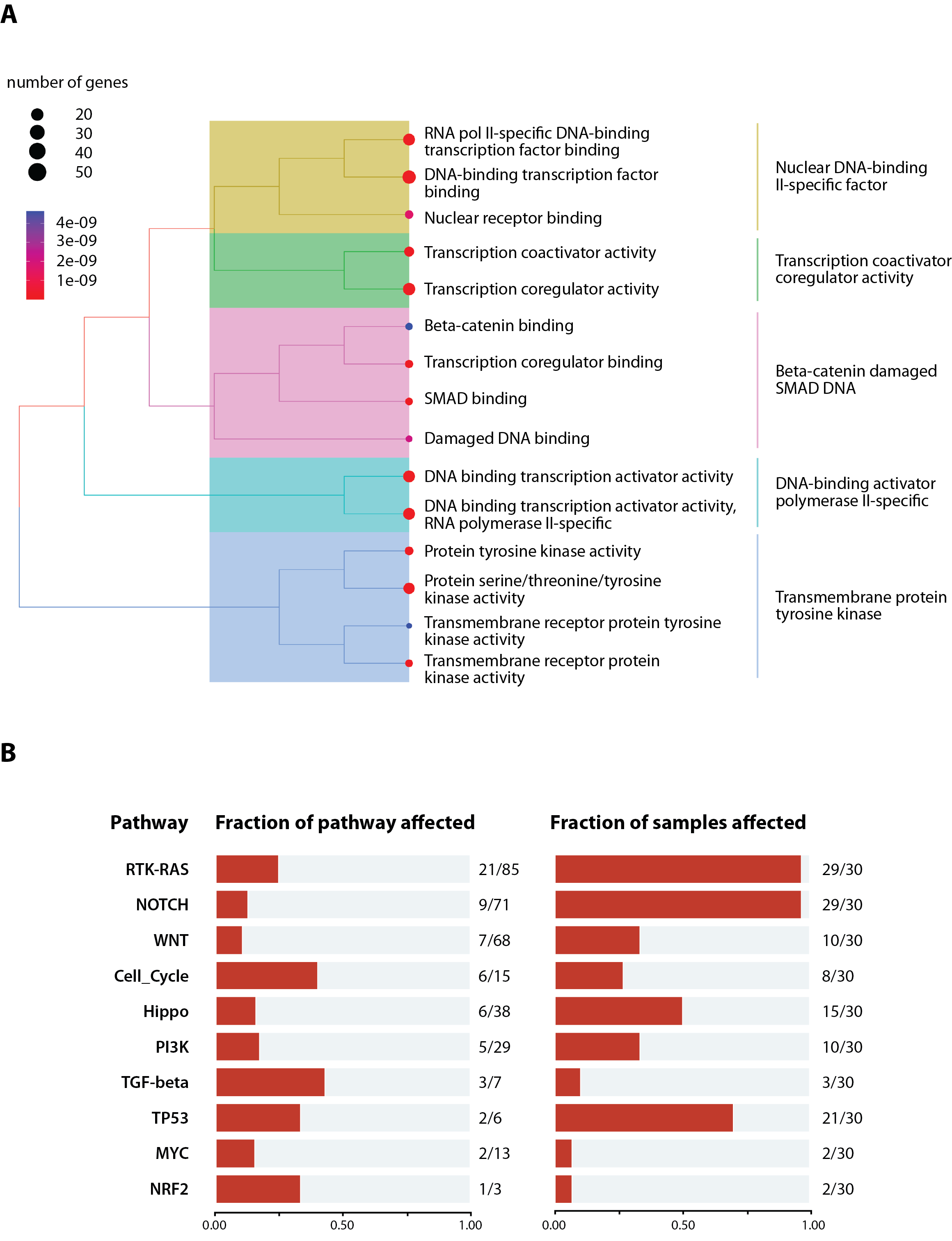

Supplement: Supplementary file 9 — Additional file 9: Figure S3. Analysis of WES from 30 human multiple myeloma cell lines. (A) Altered pathways over represented in the HMCL mutated genes. (B) mutations in oncogenic signaling pathways showingthat the RTK-RAS and NOTCH pathways are among the top mutated pathways. [file 13072_2023_497_MOESM9_ESM.png]

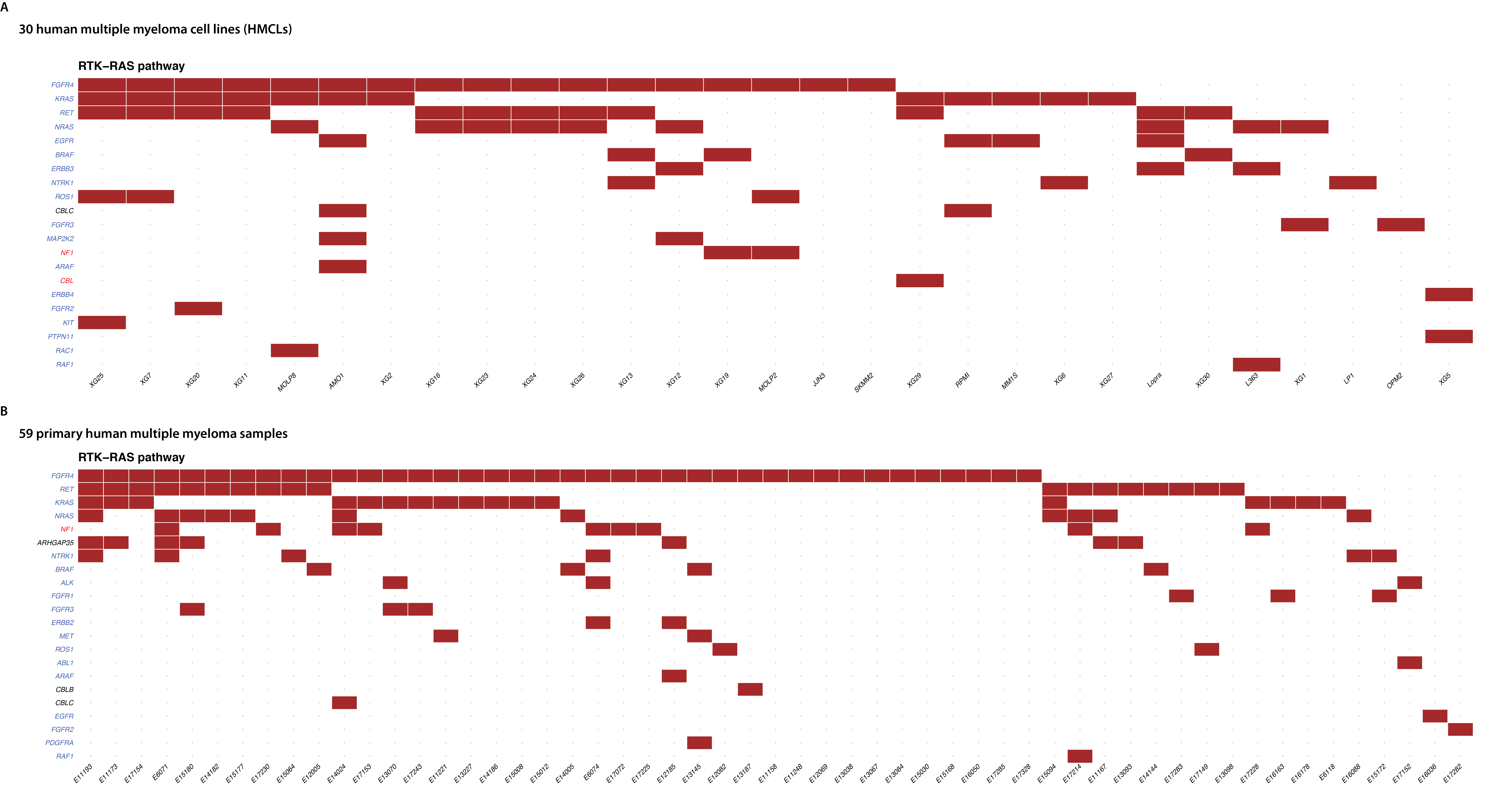

Supplement: Supplementary file 10 — Additional file 10: Figure S4. Detailed view and frequencies of the RTK-RAS pathway mutated genes. Affected genes belonging to the RTK-RAS pathway are shown and highlighted when mutated for each sample. (A)The figure shows prevalent FGFR4 (17 out of 29 samples, 58%), and KRAS (12 out of 29 samples, 41%) mutationsin HMCL, and (B) similar frequencies (FGFR4 38/59, 64%; KRAS 17/59, 28%) were observed in primary humanmultiple myeloma samples. [file 13072_2023_497_MOESM10_ESM.png]

A)

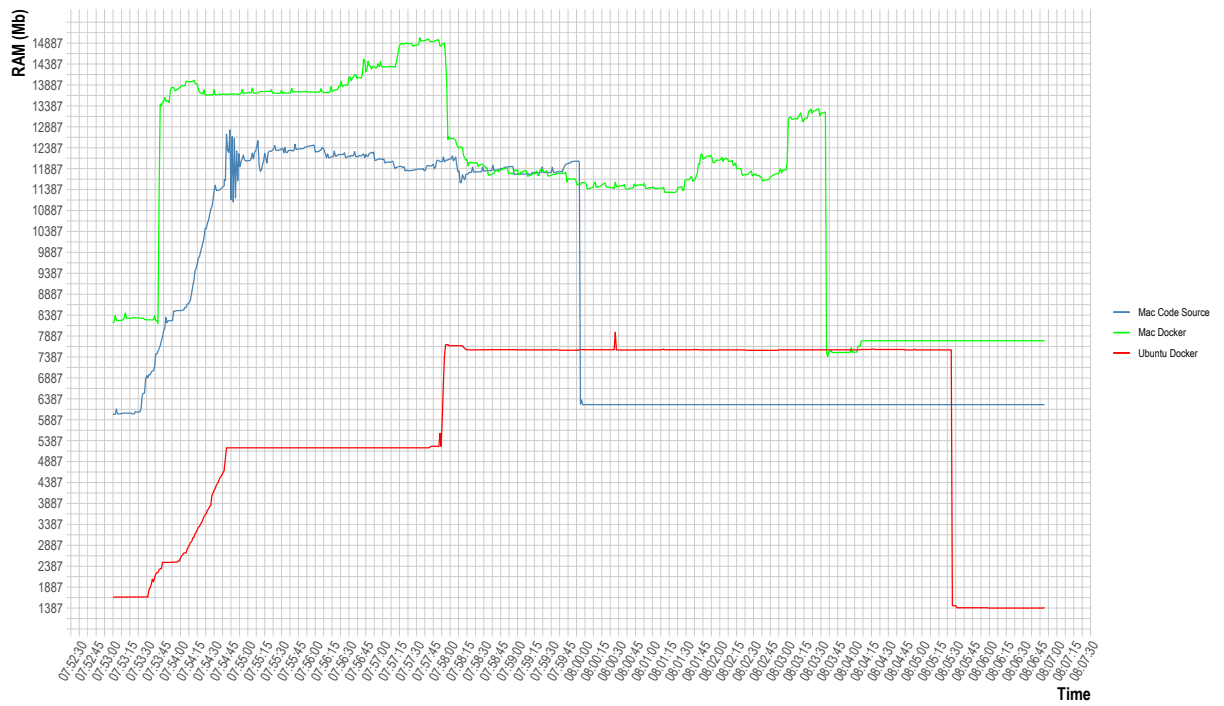

B)

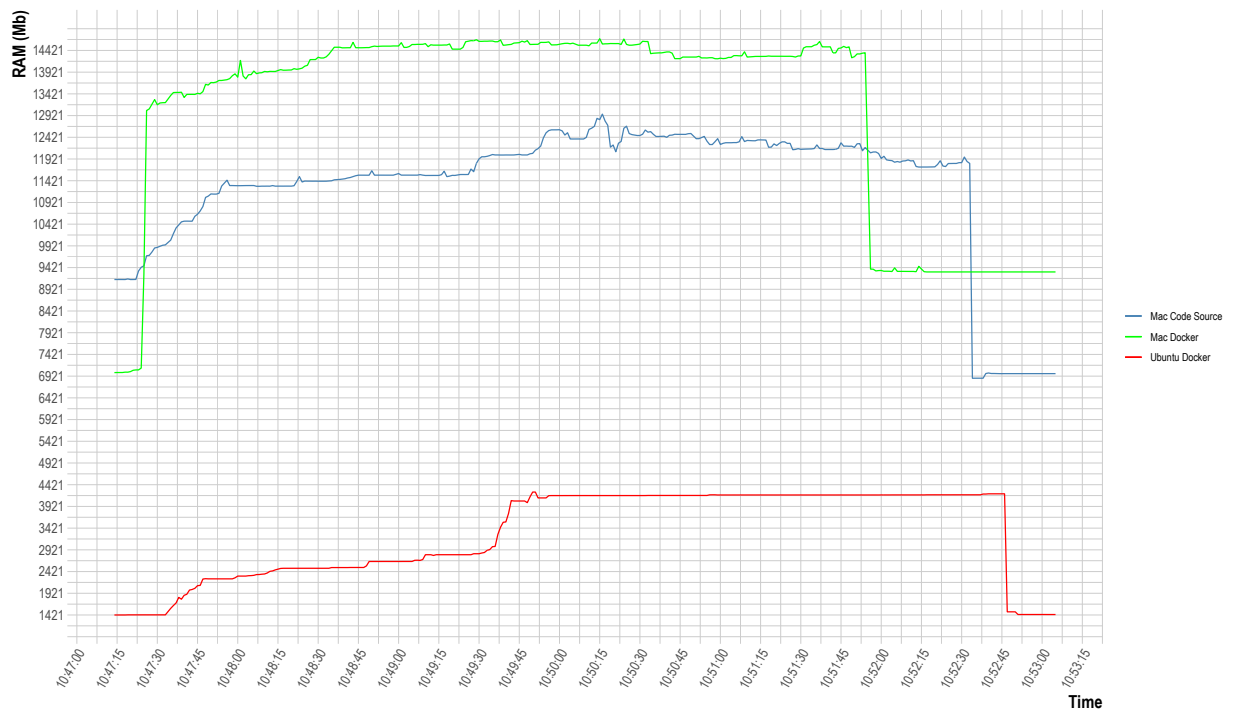

Supplement: Supplementary file 11 — Additional file 11: Figure S5. Comparison of memory resource usage of Var|Decrypt in different operatingsystems with two different methods of deployment. A) Comparison of memory resource usage during a newdata analysis. B) Comparison of memory resource usage during the reload of already analyzed data. Blue line:Var|Decrypt is installed locally on MacBook pro M1 2020 with 16GB of RAM. Green line: Var|Decrypt is deployed using docker container on a MacBook Pro M1 2020 with 16 GB of RAM. Red line: Var|Decrypt is deployed using docker container on Ubuntu (virtual machines in cloud) with 4 CPU (2GHz AMD) and 8 GB of RAM. Memory usage had been estimated using the command "ps -caxm -o rss, comm" on macOS and "ps -eo rss, comm" on ubuntu. [file 13072_2023_497_MOESM11_ESM.pdf]
